# Monochorionic Diamniotic Vasa Previa Pregnancy: A Medical Student Perspective

**DOI:** 10.1155/2021/5513139

**Published:** 2021-04-06

**Authors:** Joshua M. Samuel, Fatima Ali, Jonathan Faro, Jonathan D. Baum

**Affiliations:** Department of Obstetrics and Gynecology, Jersey Shore Medical Center, Neptune, NJ 07754, USA

## Abstract

Monochorionic diamniotic twins and vasa previa are uncommon. We present a case that was followed from ultrasound diagnosis to delivery.

## 1. Introduction

Vasa previa is a vascular pathology of the placenta where unprotected fetal vessels are in close proximity to the cervix. Labor is avoided as cervical dilation with rupture of membranes can lead to disruption of these vessels and rapid exsanguination of the fetus. The first report of this rare condition dates back to 1801, while diagnosis via ultrasound was first reported in 1987 [1].

Although uncommon, the incidence of vasa previa may be increasing due to the availability of high-quality ultrasonography [2]. Vasa previa affects up to 1 : 2500 pregnancies [3]. Twin gestation, specifically monochorionic diamniotic twins, occurs in 1 : 300 pregnancies [4]. This case is presented from a medical student perspective during subinternship at a community teaching hospital.

## 2. Case

**Figure 1:**
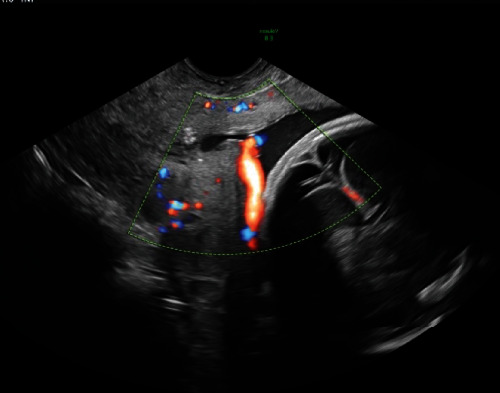
Transvaginal ultrasound image at 16 weeks showing vasa previa.

The patient is a 29-year-old G3P0111 with monochorionic diamniotic twins diagnosed with vasa previa by transvaginal ultrasound at 16-week gestation (Figure 1). Her prior pregnancy was complicated by preterm labor and delivery at 36-week gestation. The current pregnancy was otherwise uneventful, and the plan was for inpatient observation starting at 30 weeks with planned cesarean delivery to avoid labor.

At 29-week gestation, the patient presented with preterm contractions. Betamethasone was administered for fetal organ maturation, and magnesium sulfate was given for neuroprotection. She had persistent preterm contractions with cervical effacement, and therefore, cesarean delivery was indicated. During the low transverse hysterotomy, unprotected umbilical vessels were visualized. Amniotomy was performed while avoiding these vessels, and clear amniotic fluid was noted. Twin male neonates were delivered with Apgar scores 8/8 and birth weights of 1.26 kg and 1.33 kg for A and B, respectively. Both twins were evaluated and admitted to the NICU. Cord gases and hemoglobin for both twins were normal.

**Figure 2:**
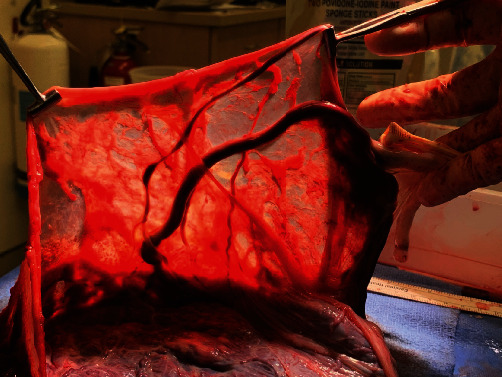
Gross specimen showing vasa previa affecting twin A.

**Figure 3:**
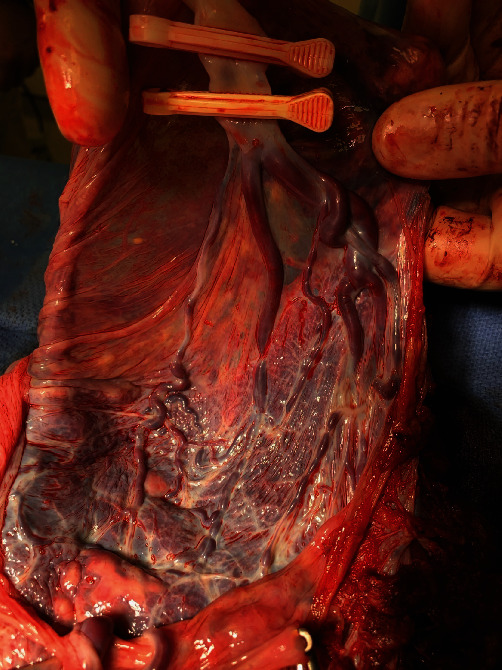
Gross specimen showing vasa previa affecting twin B.

The remainder of the surgery was uncomplicated. Gross inspection of the placenta and membranes confirmed vasa previa affecting both fetuses (see Figures 2 and 3). Placental pathology confirmed membranous insertion of the umbilical cord for twin A and twin B. The postpartum course was unremarkable, and NICU courses for infants were typical for gestational age. At the time of this writing, both twins are doing well and meeting appropriate milestones.

## 3. Discussion

Early anatomy ultrasound at 16-week gestation showed abnormal fetal vessels in relation to the placenta, consistent with vasa previa. Fetal anatomy was normal. The findings were discussed with the patient, highlighting the proximity of the fetal vessels to her cervix.

While vasa previa remains uncommon, the rate of diagnosis has increased due to the availability of high-quality ultrasonography. Furthermore, 10% of vasa previa cases are diagnosed in twin pregnancies [5].

This placental abnormality is problematic because of the location of the unprotected vessels. If the fetal head compresses these vessels, it can result in fetal hypoperfusion and acidemia [6]. Vessel rupture will lead to fetal hemorrhage, and therefore, early diagnosis is critical, highlighting the importance of ultrasound [7]. The Society of Maternal-Fetal Medicine (SMFM) recommends routine ultrasound of the lower uterine segment and the placenta during the second trimester. If vasa previa is suspected, transvaginal ultrasound with color Doppler is performed. Pulsations consistent with the fetal heart rate confirm the diagnosis [8]. Prenatal diagnosis improves outcome with neonatal survival rates up to 97%. With a postnatal diagnosis, neonatal survival decreases to less than 50% [9].

There are no guidelines on elective hospitalization for vasa previa. A recent case series showed it might be lifesaving [10]. Delivery for pregnancies complicated by vasa previa is recommended between 34 and 37 weeks to balance the risk of premature rupture of membranes, fetal hemorrhage, and death versus the risks of prematurity [11]. Given our patient's history of preterm labor and current monochorionic/diamniotic twin pregnancy, she was at increased risk for preterm delivery. Therefore, intervention on the earlier part of the recommended range was advised. The initial plan was in-hospital observation at 30-week gestation with cesarean delivery at 34 weeks.

Usually, a twin pregnancy is more than enough to excite any medical student. I remember the night this patient with twins was admitted in preterm labor. She had monochorionic diamniotic twins with vasa previa that was diagnosed in her second trimester. To the patient, everything was going as planned. To the medical student, she was a rare case that turned into a great learning opportunity. So many thoughts ran through my mind: will her contractions stop, would she hemorrhage, would the twins be okay?

Labor rooms were full, and I had “magnesium notes” to write—one of the few responsibilities delegated to medical students. I will never forget the emergency cesarean delivery that was suddenly necessary. I recalled how quickly everyone worked to get this patient into the operating room. I was immediately told to scrub in, with no time to prepare. Within a few minutes, the twins were delivered almost three months before their due date and were given immediately to the pediatric team.

**Figure 4:**
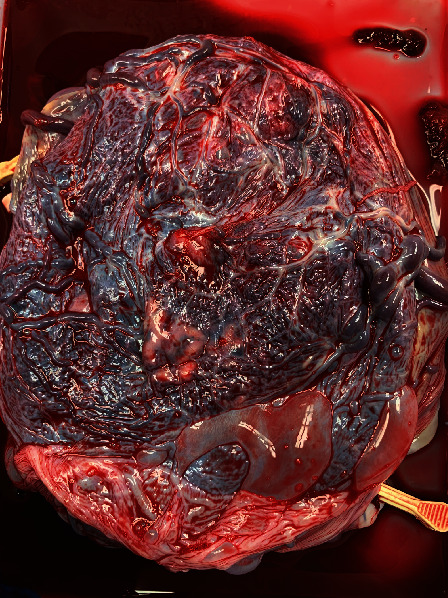
The placenta of the monochorionic diamniotic twins.

While the twins were tended to, we delivered the placenta, which appeared strikingly different from the other placentas. “Why does the placenta look like this?” I asked the attending as I continued to stare at the spongy vascular mass (Figure 4). “What you see here is called vasa previa, and this is why this patient needed surgery right away,” stated my attending (coauthor). He put the specimen in a bucket and handed it to the surgical tech, who placed it on a nearby table. As the attending started the process of “closing,” I stepped away from the operating table to take a closer look.

The vessels were large, engorged, and unprotected. It was unusual; even the OBGYN residents were fascinated. While they examined the specimen, I heard the two babies cry—a reassuring sound in any delivery room. I looked towards the warmer, where a team of pediatric residents and attendings were assessing the babies. Now, I understood why this patient needed an emergent cesarean delivery.

**Figure 5:**
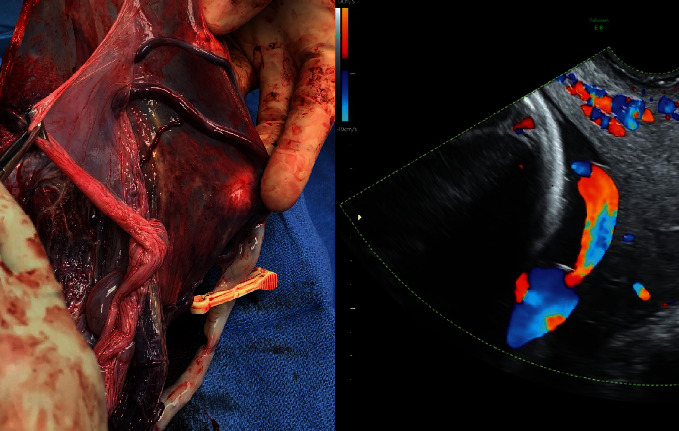
Gross image of the placenta with corresponding ultrasound. Ultrasound image shows proximity of fetal vessel to cervix.

**Figure 6:**
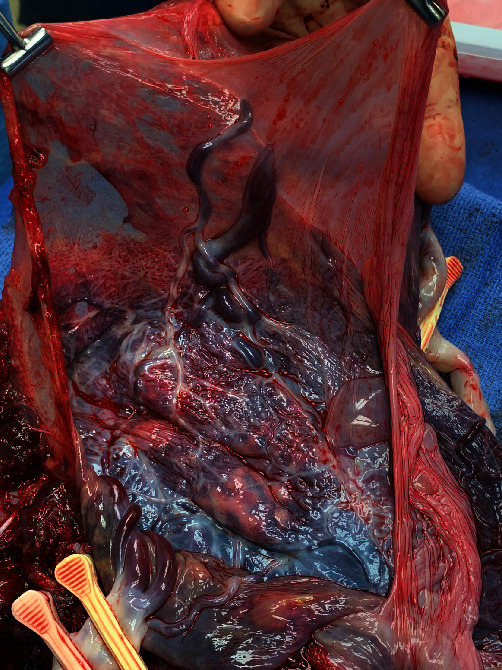
Gross image of the specimen showing vessels passing through the amnion as well as the unprotected insertion of umbilical cord.

This case highlighted the importance of ultrasound played in this patient's management. The transvaginal ultrasound with Doppler at 16-week gestation (Figure 5) illustrated the proximity of fetal vessels to the cervix. This information complicated an already high-risk pregnancy and determined the route and timing of delivery. The gross specimen shows the vessels coursing through the amnion and the unprotected insertion of the umbilical cord (Figure 6).

Reflecting on my third- and fourth-year rotations, this is the most exciting case I was a part of. The look of joy on the patient's face when she saw her twins for the first time inspired me to write this medical student perspective.

## 4. Conclusion

This paper discusses a clinical case observed during a fourth-year subinternship rotation, focusing primarily on how this patient was diagnosed with vasa previa via ultrasound, the management, and the final clinical outcome.

## Data Availability

The data presented in this case report is available from the corresponding author upon reasonable request.
